# Humoral and Cellular Response and Associated Variables Nine Months following BNT162b2 Vaccination in Healthcare Workers

**DOI:** 10.3390/jcm12093172

**Published:** 2023-04-28

**Authors:** Natalia Syrimi, Flora Sourri, Maria-Christina Giannakopoulou, Dimitrios Karamanis, Asterios Pantousas, Persefoni Georgota, Eleni Rokka, Zoe Vladeni, Euaggelia Tsiantoula, Evangelia Soukara, Nikoletta Lavda, Dimitrios Gkaragkanis, Aikaterini Zisaki, Panagiotis Vakalidis, Vasiliki Goula, Evdokia Loupou, Leonidas Palaiodimos, Dimitrios Hatzigeorgiou

**Affiliations:** 1 Paediatric Department, 251 Hellenic Air Force General Hospital, P. Kanellopoulou Avenue, 11525 Athens, Greece; 2 Infection Prevention and Control Department, 251 Hellenic Air Force General Hospital, P. Kanellopoulou Avenue, 11525 Athens, Greece; florasouri@hotmail.com (F.S.);; 3 COVID-19 Ward, 251 Hellenic Air Force General Hospital, P. Kanellopoulou Avenue, 11525 Athens, Greece; giann.maritina@gmail.com (M.-C.G.);; 4 Medical Directorate, Hellenic National and Defence General Staff, Mesogeion 227-231, 15561 Athens, Greece; dhatz57@hotmail.com; 5 Department of Health Informatics, Rutgers School of Health Professions, 65 Bergen St., Newark, NJ 07107, USA; dkaramanis@hotmail.com; 6 Department of Economics, University of Piraeus, Karaoli and Dimitriou 80, 18534 Piraeus, Greece; 7 Department of Electrical and Computer Engineering, Democritus University of Thrace, 69100 Komotini, Greece; 8 Immunology Laboratory, 251 Hellenic Air Force General Hospital, P. Kanellopoulou Avenue, 11525 Athens, Greece; 9 Oncology Ward, 251 Hellenic Air Force General Hospital, P. Kanellopoulou Avenue, 11525 Athens, Greece; 10 Biochemistry Laboratory, 251 Hellenic Air Force General Hospital, P. Kanellopoulou Avenue, 11525 Athens, Greece; 11 Department of Medicine, Jacobi Medical Center, Albert Einstein College of Medicine, New York, NY 10461, USA; leonidas.palaiodimos@gmail.com

**Keywords:** antibodies, cellular immunity, COVID-19, humoral immunity, INF-γ release assay, mRNA vaccine

## Abstract

In this study, we aimed to illustrate the trajectory of humoral and cellular immunity nine months after primary vaccination with the BNT162b2 mRNA vaccine among 189 healthcare workers (HCWs). Additionally, we endeavored to identify correlations between immunity parameters and a number of common variables and comorbidities. A total of 189 healthcare workers (HCWs), vaccinated against COVID-19, were finally included in the study. All of the subjects had received two doses of the BNT162b2 vaccine; had undergone antibody tests one, four and nine months post-vaccination; and had completed a medical questionnaire. Further samples taken at nine months were tested for cellular immunity. No participants had evidence of COVID-19 infection pre- or post-vaccination. An anti-S1 receptor binding domain (RBD) antibody assay was used to assess humoral response, and cellular immunity was estimated with an INF-γ release assay (IGRA). Statistical analysis was performed using STATA. We report a statistically significant antibody drop over time. Being above the age of 40 or a smoker reduces the rise of antibodies by 37% and 28%, respectively. More than half of the participants did not demonstrate T-cell activation at nine months. Female gender and antibody levels at four months predispose detection of cellular immunity at nine months post-immunization. This study furthers the qualitative, quantitative, and temporal understanding of the immune response to the BNT162b2 mRNA vaccine and the effect of correlated factors.

## 1. Introduction

The necessity to contain the COVID-19 pandemic impelled the emergency authorization of novel mRNA vaccines. The BNT162b2 mRNA vaccine has been administered to billions of people worldwide with a two-dose schedule proven to be 95% effective for preventing severe COVID-19 disease caused by wild-type virus and several mutations [1,2,3,4,5]. BNT162b2 has demonstrated a high efficacy rate even against variants of concern and has an acceptable safety profile [6]. Nevertheless, the decline of antibody levels post vaccination along with the increasing numbers of breakthrough infections among vaccinated individuals [7,8,9] has created uncertainty about the durability of protective immunity and has necessitated serial booster doses for the adult population. 

The rise of specific antibodies against SARS-CoV-2 after natural infection or vaccination has been widely examined [10,11,12,13,14]. Evidence is scarce regarding the question as to whether these antibodies directly correlate with protection or constitute at least one of the protective immune mechanisms [15]. A large UK study (the SIREN study) has suggested that natural infection and induction of antibody response provides robust protection against asymptomatic and symptomatic reinfection [10]. Similarly, studies have demonstrated that available vaccines are able to elicit a significant humoral response in vaccinees with a peak antibody level measured one month after immunization [11,16,17,18]. Previous natural COVID-19 infection is associated with higher levels of humoral response in BNT162b2 mRNA vaccinated individuals, enabling hybrid immunity to promise long-term protection [19,20]. 

However, the rise of antibody titers per se is not necessarily associated with protection and the level above which we consider the antibodies to be protective is yet to be validated [21,22,23,24]. Conversely, the observation that antibody titers wane over time [21,25,26,27,28,29,30,31,32,33] has raised concerns regarding the level of residual protection and shifted the focus of scientific inquiry to other correlates of immunity to more accurately assess protection.

Vaccines are able to confer immunity by targeting not only the humoral but also the cellular branch of the immune system [34,35]. There is mounting evidence that T-cell response is elicited both in naturally infected patients and vaccinated individuals and can provide long-term protection [36,37,38,39,40,41,42,43,44,45,46,47,48,49]. Nevertheless, the trajectory of long-term antigen-specific T-cell response following mRNA vaccination remains incompletely investigated. Cellular assays are expensive and time-consuming and require experienced lab personnel to execute. Other methods that indirectly assess cellular response, such as interferon gamma release assays (IGRA), are emerging in the literature as both sensitive and accurate in assessing T-cell antigen-specific responses in cohorts of SARS-CoV-2 convalescent and vaccinated populations [50,51,52,53,54]. 

The most important risk factors for serious disease from SARS-CoV-2 are old age and the presence of comorbidities [27,29,30,55,56]. Male gender, smoking, and obesity are also well-established factors for worse outcomes [57,58,59]. According to the literature, the efficacy of the BNT162b2 vaccine against SARS-CoV-2 could be correlated with the above characteristics with a difference in elicited humoral responses [60,61,62,63,64].

This study aims to elucidate aspects of the humoral and cellular response to vaccination with the BNT162b2 vaccine, and to assess the magnitude and the longitude of antibody titers measured up to nine months post-vaccination.

## 2. Materials and Methods

### 2.1. Population and Study Design

**Figure 1:**
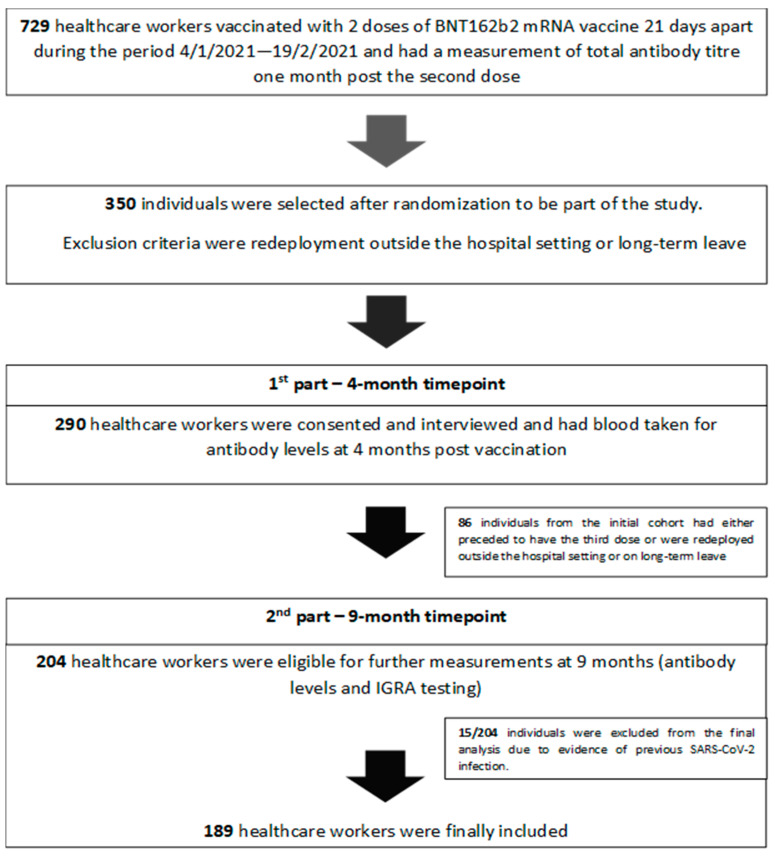
Study design.

This is a single-centered, prospective, longitudinal study conducted at 251 Air Force General Hospital, in Athens. Seven hundred and twenty-nine (729) healthcare workers (HCW) were vaccinated with two doses of the BNT162b2 mRNA vaccine 21 days apart during the period of 4 January 2021–19 February 2021, when a mass vaccination campaign was initiated in-house for hospital staff. The workers all had a measurement of total antibody titers one month after receiving the second dose as per a hospital offer to check for immune response. Out of 729 individuals eligible for inclusion, 350 were selected after randomization to form our initial sample population. (Figure 1). 

### 2.2. Data Extraction

The sample population was contacted in person or by telephone by study investigators and written informed consent was obtained before enrollment. Out of 350 individuals randomly selected, 290 were consented and interviewed. Data were collected using questionnaires to investigate demographic features (age and gender), anthropometric data (weight and height), smoking habits, and past medical history/regular medications. Body mass index (BMI) was calculated as weight in kilograms divided by squared height in meters (kg/m^2^). Morbidity recorded was classified into further subgroups including diabetes mellitus, lung disease (asthma, chronic obstructive pulmonary disease, pulmonary fibrosis), chronic renal failure, heart disease (coronary heart disease, myocardiopathy, heart failure), hypertension, dyslipidemia, immunosuppression (cancer or immunosuppressive treatment), autoimmunity.

### 2.3. Blood Collection Four and Nine Months Post Full Vaccination

Blood collection was scheduled four months after the second inoculation with a maximum delay of 10 days. A total of 290 blood samples were sent to the biochemistry lab for centrifuge and quantification of post-vaccination antibody levels. Samples collected were kept in the refrigerator (between +2 °C and +8 °C) and were analyzed within seven days. At the nine-month timepoint, estimation of long-term antibody levels in addition to T-cell activation profile was attempted. Of 290, 204 individuals were eligible for the second phase of the study (Figure 1), and 204 paired blood samples were analyzed. In addition to biochemistry samples sent for antibody levels, another 5 mL of whole blood from each participant (collected in five lithium heparin tubes (1 mL each)) were sent to the lab for IGRA testing. Whole blood was harvested after 16–24 h of stimulation at 37 °C and then assessed for IFN-γ.

### 2.4. Laboratory Methods

#### 2.4.1. Anti-SARS-CoV-2 Antibodies

Antibody levels were measured in serum samples using the ADVIA Centaur^®^ SARS-CoV-2 IgG (sCOVG) (Siemens Healthcare Diagnostics Inc., Tarrytown, NY, USA) assay, a quantitative chemiluminescence immunoassay that uses the receptor binding domain (RBD) of the spike protein 1 as capture antigen. All samples were processed according to the manufacturer’s instructions using an automated platform (ADVIA Centaur^®^ XP systems, Siemens (Siemens Healthineers, Erlangen, Germany)) and yielded results with 96.41% sensitivity and 99.9% specificity. A result of reactive or nonreactive was determined according to the index value established with the calibrators. A cut-off level of 1 U/mL determined a positive result. 

#### 2.4.2. IGRA

T-cell activation was evaluated using a COVI-FERON kit (SD Biosensor, Inc., Cheongju-si, Republic of Korea), an IGRA approved for use in in vitro diagnosis (IVD). The assay consists of five antigen tubes aiming to stimulate T-lymphocytes involved in cell-mediated immunity in heparinized whole blood. Nil tube estimates the background IFN-γ level of the sample. Original spike protein (OSP) antigen tube assesses the IFN-γ responses to SARS-CoV-2 spike protein (SP) antigen derived from the wild-type virus (Wuhan) and 20I/501Y.V1 (UK) variant. Variant spike protein (VSP) antigen tube assesses the IFN-γ responses to SARS-CoV-2 SP antigen derived from the 20H/501.V2 (South Africa) and 20I/501Y.V3 (Brazil) variants. A mitogen tube is used as positive control. NP Antigen tube is used to speculate IFN-γ responses to SARS-CoV-2 nucleocapsid protein (NP) antigen indicative of previous natural COVID-19 infection. Plasma from the stimulated samples was used for detection of INF-γ production using an enzyme-Linked immunosorbent assay (ELISA)-based platform. Specimens were processed as per the manufacturer’s advice. Quantitative results (INF-γ concentration in IU/mL) were recorded and further analyzed. An elevated response was defined as a value greater than at least 0.3 IU/mL, implying detectable cellular immunity with 97% sensitivity and 94.2% specificity. Finally, results were appropriately modified to represent the index and >1 was considered positive. 

### 2.5. Ongoing Disease Surveillance

A significant benefit of all the study subjects being members of the hospital staff was that it enabled in-house surveillance for disease incidence throughout the study via several modalities: biweekly nasopharyngeal testing in high exposure placements, prompt reporting of clinical signs and symptoms and immediate testing, and close tracking and tracing of index cases and high-risk exposures. As a result, before the final analysis, 15 participants were further excluded due to evidence of COVID-19 infection (Figure 1). This was evidenced either by positive PCR or by a positive result for previous natural infection in IGRA testing (NP index). A total of 189 samples remained eligible for analysis of humoral and cellular response in COVID-19 naïve individuals. At the time of the study, the delta variant had emerged and was responsible for the main burden of infections.

### 2.6. Statistical Analysis

Participants were divided into two age groups (≤40 and >40 years old), and three BMI subgroups (BMI: <25 kg/m^2^ (Underweight/Normal), BMI: 25–29.9 kg/m^2^ (Overweight), BMI: ≥30 kg/m^2^ (Obese/Extremely Obese)). They were further classified according to their status of original SP and variant SP cellular immunity (no/yes). For descriptive analysis, continuous variables are presented as median with interquartile range (IQR) and categorical ones as absolute and relative frequencies. The antibody titers from the first, fourth, and ninth month are also presented as means with standard deviation, and the difference between the three measurements is accessed through a repeated-measures ANOVA test. Differences between the groups were compared with *t*-test or Mann–Whitney test for the continuous data and the chi-squared or Fisher exact test for the categorical variables, while the Shapiro–Wilk test was used to access the normality assumption of the data.

Mixed linear regression models were used to identify baseline characteristics associated with antibody titers. The natural logarithm (ln) of the antibody titers was used as the independent variable, and four multivariate models are presented for added robustness. Model 1 refers to the repeated antibody titer measurements for months 1 to 4, model 2 refers to months 4 to 9, model 3 refers to months 1 to 9, and model 4 also to months 1 to 9 including an interaction term of the variables with a significant univariate association (*p*-value < 0.05), that is, the age group and smoking status.

Logistic regression analysis was performed to detect factors that might be associated with original SP and variant SP cellular immunity. Antibody titers were evaluated both in their original and ln-transformed scale. Two multivariate models are presented, the first includes the antibody titers in their original scale, while the second represents their ln-transformed scale.

Statistical analysis was performed with STATA and a two-tailed *p*-value < 0.05 was considered significant.

## 3. Results

One hundred eighty-nine (189) HCWs were ultimately included in this analysis. The descriptive characteristics of the study group are summarized in Table 1. All participants were of Caucasian ethnicity, their median age was 43 years old (range: 36–50) and 48.7% (*n* = 92) of them were male. Additionally, 37.6% (*n* = 71) were younger than 40 years old, 39.2% (*n* = 74) and 14.8% (*n* = 28) were overweight and obese respectively, and 31.8% (*n* = 60) were current smokers. Moreover, 7.4% (*n* = 14) reported being hypertensive on medication, 5.8% (*n* = 11) reported having dyslipidemia, 9% (*n* = 17) endorsed autoimmunity (7 of them referred Hashimoto), and 2.1% (*n* = 4) claimed to have some degree of immunosuppression.

Mean antibody levels at one, four, and nine months after the second dose of BNT162b2mRNA were 153.49 U/mL, 32.38 U/mL, and 19.65 U/mL respectively. All participants had detectable antibodies (>1 U/mL) one and four months after their second dose but 7/204 (3.4%) dropped their antibody levels to less than 1 U/mL at nine months.

A 78.9% decline in median antibody levels was calculated between the first and fourth month, and a 39.31% decline between the fourth and ninth month, revealing a continued reduction, albeit at a slower pace. ANOVA test for repeated measurements was used and corroborated a significant time effect for the mean antibody level kinetics (*p* < 0.001). A post hoc pairwise comparison using the Bonferroni correction revealed a statistically significant drop in antibody levels between one and four months (*p* < 0.001), but the drop was not statistically significant between the timepoints of four and nine months (*p* = 0.458). 

To determine whether vaccine-induced antibody responses depended on sex, age, BMI, or specific comorbidities, we investigated the induction in antibodies one, four, and nine months after the second vaccine dose concerning these variables (Table 2). We sought to examine which characteristics might be significantly associated with antibodies at their peak detection concentration one month after the second dose. Younger participants (<40 years old) had significantly higher antibody levels at one month (mean 172 (106–210), *p* = 0.003), at four months (mean 32 (17–52), *p* < 0.001), and at nine months after a second dose (mean 5.66 (3.45–8.65), *p* < 0.001) than older participants. Moreover, the concentration of antibodies was increased in the non-smoker group at the first month (mean 159 (100–195), *p* = 0.009), at four months (mean 24 (13–49), *p* < 0.001), and at nine months after the second dose (mean 4.78 (2.84–8.22), *p* < 0.001) compared with smokers (Table 2). COVI-FERON ELISA assay demonstrated that, nine months after the second dose, the IFN-γ concentration against the original SARS-CoV-2 spike protein (OSP) was positive in 43.9% (*n* = 83) and the IFN-γ concentration against the variant SARS-CoV-2 spike protein (VSP) was positive in 27% (*n* = 51) (Table 1).

### 3.1. Mixed Linear Regression Analysis

**Figure 2:**
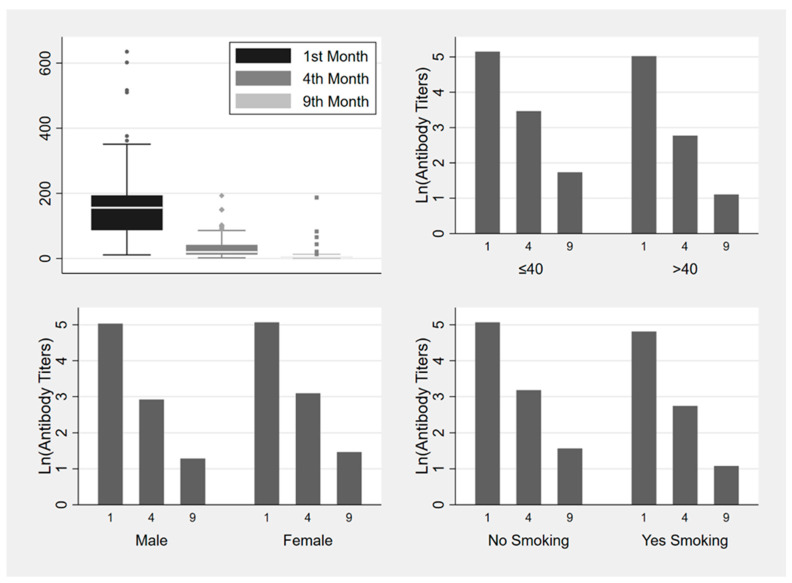
Box plot and bar plots (median values) of ln (antibody titers) variance one, four and nine months after the second dose of BNT162b2 vaccine and risk factors (age, gender, smoking status).

To investigate the possible correlation of vaccine-induced antibody responses with age, sex, BMI, smoking habits, or specific comorbidities, we conducted univariate and multivariate mixed linear model analyses. Older age (>40) and smoking habit were steadily associated with lower antibody levels in measurements at all three of the time spans (one to four months (*p* = 0.001, *p* = 0.03), four to nine months (*p* < 0.002, *p* = 0.007), and one to nine months (*p* = 0.002, *p* = 0.029)) and these correlations were statistically significant (Figure 2, Table 3, models 1–3). BMI, sex, hypertension, dyslipidemia, immunosuppression, and autoimmune disease were not significantly associated to antibody levels in any case. 

Mixed linear model analysis showed that being above the age of 40 or being a smoker reduces the development of antibodies by 37% (β Estimate: −0.466, CI: −0.765–−0.167, *p*-value = 0.002) and 28% (β Estimate: −0.328, CI: −0.623–−0.033, *p*-value = 0.029) respectively, during the first nine months after vaccination (Table 3—model 3). The interaction effect between the two variables was also found to be significant. Specifically, it was revealed that being above the age of 40 and a smoker reduces the development of antibodies by 55% (β Estimate: −0.796, CI: −1.177–−0.416, *p*-value < 0.001) (Table 3—model 4).

### 3.2. Original SP Index

The univariate associations with the original SP index of cellular immunity were examined for all baseline demographic data, clinical characteristics, and antibody levels at one, four, and nine months on the original and ln-transformed scale. Female gender and antibody levels at one and four months on the original scale, and ln-transformed antibody levels for all months were found to have a significant univariate association as opposed to age, underlying conditions, and antibody levels at nine months (original scale) (Table 4, Part A). In the multivariable analysis, only in model 1, female gender (OR: 0.477; 95% CI: 0.238–0.956; *p* = 0.037) and antibody levels at four months (OR: 1.016; 95% CI: 1.002–1.031; *p* = 0.028) were found to be significantly associated with the presence of original SP index cellular immunity at nine months post-vaccination (Table 4, Part A). Results are similar if we account for the standardized or the normalized transformed scales of the antibody levels.

### 3.3. Variant SP Index

Antibody levels at four months in their original scale and antibody levels at four and nine months in their ln-transformed scale were found to have a significant univariate association with the VSP index of cellular immunity (Table 4, Part B). In the multivariate analysis for the VSP index, none of the examined parameters were found to have a significant association (Table 4, Part B).

## 4. Discussion

In our study, anti-RBD antibody levels are observed to drop over nine months following vaccination, yet remain detectable in most cases; findings that are in agreement with other studies [31,65,66,67,68,69]. In our cohort of 189 HCWs, antibody decay was estimated to be 78.9% between one and four months after the second dose, and a further 39.3% between four and nine months (Figure 2). Similar antibody trajectories derive from other studies where the anti-S-RBD antibody fall rate six months after the initial vaccination scheme is estimated to range between 60–90% [65,69].

Several studies provide reassuring evidence that robust and long-lasting activation of spike-specific T-cells takes place and can outweigh humoral response as the main indicator of vaccine effectiveness [70,71,72]. Malipiero et al. have demonstrated that IGRA can be used as an accurate laboratory method to estimate cellular immune response to BNT162b2 mRNA vaccine in both immunocompetent and immunocompromised patients where the humoral response is undetectable [52]. Tychala et al. found a positive correlation of antibody levels with INF-γ-based cellular response five months post BNT162b2 vaccination [73]. 

In our study, IGRA estimation of cellular immunity nine months post vaccination shows an active cellular response to original SP in 83 participants (44%) and variant SP in 51 (26%), meaning that more than half of the participating individuals lose their highly desired cellular response by nine months (Table 1). Interestingly, we report a positive association of original SP cellular immunity with female sex and antibody levels at four months that was well-supported through multivariate regression analysis (Table 1). These findings have not been previously reported. Similar correlations are not found for cellular immunity as a response to variant SP. 

A clear negative correlation is noted between increasing age and antibody titers at one, four, and nine months post vaccination, which is in line with other studies [21,31,69,74,75,76,77,78,79]. This correlation is persistent in all three measurements and is validated with logistic regression analysis and a mixed linear model. Other studies also corroborate an age-related impairment of binding and neutralizing antibodies after vaccination [80,81,82], while cellular immunity does not appear to be affected by age in our study or elsewhere [83,84,85]. We also report that being a smoker weakens antibody development by 37%, whereas being above the age of 40 and a smoker reduces the development of the antibodies by 55%. Likewise, Nomura et al. found that age and smoking habit determine antibody response three months post receipt of second vaccine [79], and the VASCO study describes a rapid decrease in antibody levels post BNT162b2 mRNA vaccine among smokers [83].

Conflicting data in the literature demonstrate either an increased capacity for females to mount humoral immune responses compared with males [62,85,86,87,88,89], or do not associate gender with antibody response [56,69,90,91,92]. In our study, we did not find an association between the female gender and antibody levels post full immunization, but we report a correlation between the female gender and the presence of activated anti-S T-cells at nine months post-immunization [31,65,93,94].

Studies have shown that mRNA COVID-19 vaccines had similar efficacy among obese and non-obese individuals [1,16,89], while others report a decreased antibody response to the first dose of BNT162b2 COVID-19 vaccine in the obesity or pre-obesity group [62,95]. In the present study, BMI is not found to significantly impair antibody or cellular response. Watanabe et al. have correlated central obesity (defined as higher waist circumference) as a factor negatively affecting the post-vaccination development of antibodies [56]. Alternatively, several studies suggest that obese individuals develop surprisingly higher neutralizing antibodies compared with their recipients with normal weight [31,96]. 

Regarding the effects of comorbidity, findings from several studies indicate blunted post-vaccination humoral response in people with diabetes [17,56], hypertension [56,89], dyslipidaemia [56], immunosuppression [97], autoimmune diseases and heart disease [66,93,98]. In our study, no association is found between humoral or cellular immunity and underlying medical conditions, possibly because of the small number of participants with comorbidities in this cohort. It should be acknowledged that this is a limitation of our study.

This is a single-centered study, yet the size of the sample is relatively high compared with others published so far. Another limitation of the study is that there is no baseline sample collected before vaccination and COVID-19 negative status was determined by infection surveillance. Moreover, the study population in this report is relatively young and healthy without a significant burden of comorbidities. Therefore, the generalizability of our results may not be taken for granted. Demographic and anthropometric data in this study were obtained via a standardized structured self-reporting questionnaire, introducing the possibility of recall bias. Additionally, cell-mediated immunogenicity was only measured at nine months post-vaccination, as our country’s kit for cellular immunity was not commercially available earlier. The company we sourced it from lists the date of registration in their database as 24 May 2021. In our study, vaccinees with previous COVID-19 have been excluded, thus our measurements of humoral and cellular immunity reflect the duration of vaccine-induced immunity in COVID-19 naive population. The limitations of this study prevent us from drawing definitive conclusions but do add to existing and future scientific data.

## 5. Conclusions

The long-term effectiveness of the primary vaccination scheme of mRNA vaccines is unknown and results from large-scale trials are warranted to indicate the optimal vaccination strategy against SARS-CoV-2 and the necessity of serial booster doses. This study provides insights into the evolution, duration, and associated factors of vaccine-induced immunity—both humoral and cellular—long after the completion of the two-dose BNT162b2 vaccine schedule.

## Figures and Tables

**Table 1:** Baseline demographics, antibody measurements and cellular immunity.

			Cellular Immunity Original Spike Protein			Cellular Immunity Variant Spike Protein		
	Initial Sample	Final Sample	Inactive	Active		Inactive	Active	
Characteristic	*n* = 290	*n* = 189	*n* = 106	*n* = 83	*p*-Value	*n* = 138	*n* = 51	*p*-Value
Age—median (IQR)	44.00 (36.00–51.00)	43.00 (36.00–50.00)	44.00 (36.00–51.00)	43.00 (35.00–50.00)	0.436	44.00 (36.00–50.00)	41.00 (35.00–51.00)	0.508
Age Category-no. (%)								
≤40	102 (35.17)	71 (37.57)	36 (33.96)	35 (42.17)	0.248	48 (34.78)	23 (45.10)	0.194
>40	188 (64.83)	118 (62.43)	70 (66.04)	48 (57.83)		90 (65.22)	28 (54.90)	
Gender								
Male	145 (50.00)	92 (48.68)	45 (42.45)	47 (56.63)	0.053	65 (47.10)	27 (52.94)	0.476
Female	145 (50.00)	97 (51.32)	61 (57.55)	36 (43.37)		73 (52.90)	24 (47.06)	
BMI-median (IQR)	25.35 (22.50–28.65)	25.35 (22.50–28.40)	25.12 (22.46–28.37)	25.86 (22.84–28.69)	0.492	25.20 (22.46–28.40)	25.88 (23.51–28.65)	0.762
BMI Category—no. (%)								
Underweight/Normal	134 (46.21)	87 (46.03)	52 (49.06)	35 (42.17)	0.599	66 (47.83)	21 (41.18)	0.713
Overweight	104 (35.86)	74 (39.15)	40 (37.74)	34 (40.96)		52 (37.68)	22 (43.14)	
Obese/Extreme Obese	52 (17.93)	28 (14.81)	14 (13.21)	14 (16.87)		20 (14.49)	8 (15.69)	
Smoking—no. (%)								
No	195 (67.24)	129 (68.25)	68 (64.15)	61 (73.49)	0.171	92 (66.67)	37 (72.55)	0.441
Yes	95 (32.76)	60 (31.75)	38 (35.85)	22 (26.51)		46 (33.33)	14 (27.45)	
Hypertension—no. (%)								
No	268 (92.41)	175 (92.59)	99 (93.40)	76 (91.57)	0.634	130 (94.20)	45 (88.24)	0.164
Yes	22 (7.59)	14 (7.41)	7 (6.60)	7 (8.43)		8 (5.80)	6 (11.76)	
Immunosuppression—no. (%)								
No	282 (97.24)	185 (97.88)	103 (97.17)	82 (98.80)	0.441	135 (97.83)	50 (98.04)	0.928
Yes	8 (2.76)	4 (2.12)	3 (2.83)	1 (1.20)		3 (2.17)	1 (1.96)	
Dyslipidemia—no. (%)								
No	265 (91.38)	178 (94.18)	100 (94.34)	78 (93.98)	0.916	130 (94.20)	48 (94.12)	0.982
Yes	25 (8.62)	11 (5.82)	6 (5.66)	5 (6.02)		8 (5.80)	3 (5.88)	
Autoimmune—no. (%)								
No	262 (90.34)	172 (91.01)	96 (90.57)	76 (91.57)	0.811	124 (89.86)	48 (94.12)	0.363
Yes	28 (9.66)	17 (8.99)	10 (9.43)	7 (8.43)		14 (10.14)	3 (5.88)	
Antibody Measurements								
1st month								
Median (IQR)	154.50 (69.00–189.00)	156.00 (77.00–194.00)	154.50 (61.00–188.00)	160.00 (100.00–201.00)	0.031	155.50 (73.00–191.00)	164.00 (114.00–195.00)	0.082
Mean (sd)	146.08 (100.16)	153.49 (97.24)	140.52 (91.15)	170.05 (102.68)		146.63 (94.35)	172.04 (103.33)	
4th month								
Median (IQR)	18.50 (11.00–38.00)	20.00 (12.00–41.00)	18.00 (11.00–34.00)	26.00 (13.00–53.00)	0.007	19.00 (11.00–39.00)	26.00 (13.00–60.00)	0.038
Mean (sd)	32.45 (37.52)	32.38 (32.22)	26.26 (25.33)	40.19 (38.07)		29.08 (28.71)	41.31 (39.18)	
9th month								
Median (IQR)	-	3.98 (2.46–6.95)	3.48 (2.38–5.99)	5.49 (2.80–8.82)	0.058	3.59 (2.31–6.40)	5.38 (2.87–8.44)	0.069
Mean (sd)		19.65 (109.17)	6.37 (18.36)	36.61 (162.40)		10.89 (56.09)	43.37 (188.17)	
INDEX OSP	-	0.72 (0.33–1.95)	0.36 (0.22–0.52)	2.14 (1.53–3.11)	<0.001	0.46 (0.26–1.03)	2.47 (1.74–4.75)	<0.001
INDEX VSP	-	0.43 (0.17–1.15)	0.19 (0.10–0.38)	1.24 (0.72–1.95)	<0.001	0.24 (0.12–0.47)	1.79 (1.41–2.88)	<0.001
INDEX Nucleocapsid Protein	-	0.04 (0.00–0.09)	0.05 (0.02–0.11)	0.00 (−0.04–0.07)	0.001	0.04 (0.01–0.09)	0.01 (−0.03–0.09)	0.715
Cellular immunity OSP—no. (%)								
No	-	106 (56.08)	106 (100.00)	0 (0.00)	<0.001	103 (74.64)	3 (5.88)	<0.001
Yes	-	83 (43.92)	0 (0.00)	83 (100.00)		35 (25.36)	48 (94.12)	
Cellular immunity VSP—no. (%)								
No	-	138 (73.02)	103 (97.17)	35 (42.17)	<0.001	138 (100.00)	0 (0.00)	<0.001
Yes	-	51 (26.98)	3 (2.83)	48 (57.83)		0 (0.00)	51 (100.00)	

Notes: *p*-values refer to Mann–Whitney test or independent-samples *t*-test for the continuous variables and Chi-squared or Fisher exact test for the categorical data. BMI in kg/m^2^. Abbreviations: BMI = body mass index, IQR = interquartile range, no. = number, OSP = original spike protein, VSP = variant spike protein.

**Table 2:** Antibody values at one, four, and nine months after immunization and risk factors.

Characteristic	First Month—Median (IQR)	*p*-Value	Fourth Month—Median (IQR)	*p*-Value	Ninth Month—Median (IQR)	*p*-Value
Age Category						
≤40	172.00 (106.00–210.00)	0.003	32.00 (17.00–52.00)	<0.001	5.66 (3.45–8.65)	<0.001
>40	151.50 (67.00–184.00)		16.00 (10.00–33.00)		3.01 (1.92–6.30)	
Gender						
Male	153.50 (75.50–192.50)	0.425	18.50 (11.50–33.00)	0.151	3.60 (2.68–6.31)	0.515
Female	159.00 (84.00–198.00)		22.00 (12.00–52.00)		4.32 (2.34–8.25)	
BMI Category						
Underweight/Normal	156.00 (87.00–202.00)	0.877	22.00 (13.00–41.00)	0.372	4.32 (2.67–7.51)	0.086
Overweight	155.50 (69.00–191.00)		18.00 (11.00–42.00)		3.49 (2.26–6.89)	
Obese/Extreme Obese	161.00 (80.50–198.50)		24.00 (13.00–43.50)		3.59 (2.30–6.80)	
Smoking						
No	159.00 (100.00–195.00)	0.009	24.00 (13.00–49.00)	<0.001	4.78 (2.84–8.22)	<0.001
Yes	123.00 (58.00–184.00)		15.50 (10.00–29.50)		2.94 (1.61–5.54)	
Hypertension						
No	156.00 (77.00–194.00)	0.915	20.00 (12.00–42.00)	0.564	3.98 (2.53–7.14)	0.581
Yes	156.50 (61.00–212.00)		20.50 (7.00–35.00)		3.58 (1.37–6.95)	
Immunosuppression						
No	156.00 (77.00–194.00)	0.494	20.00 (12.00–41.00)	0.413	3.98 (2.46–6.95)	0.732
Yes	179.00 (126.00–196.50)		44.50 (13.50–109.50)		5.08 (2.36–10.79)	
Dyslipidemia						
No	156.00 (74.00–194.00)	0.681	20.00 (12.00–41.00)	0.705	3.97 (2.53–6.95)	0.842
Yes	156.00 (94.00–224.00)		20.00 (10.00–69.00)		4.93 (2.00–7.51)	
Autoimmune						
No	156.00 (80.00–194.50)	0.488	21.00 (12.00–43.50)	0.172	4.16 (2.50–7.45)	0.245
Yes	154.00 (77.00–180.00)		18.00 (9.00–28.00)		3.02 (2.23–5.78)	

Note: *p*-values refer to Mann–Whitney test. Abbreviations: BMI = body mass index, IQR = interquartile range.

**Table 3:** Antibody kinetics dependent on time and the dependent variables (mixed linear regression).

	Univariate Mixed Linear Model	Multivariate Mixed Linear Model			
		(1)	(2)	(3)	(4)
	*n* = 567	*n* = 378	*n* = 378	*n* = 567	*n* = 567
	Months 1 to 9	Months 1 to 4	Months 4 to 9	Months 1 to 9	Months 1 to 9
Dependent Variable: Ln (Antibodies)	β Estimate, 95% CI, *p*-Value	β Estimate, 95% CI, *p*-Value	β Estimate, 95% CI, *p*-Value	β Estimate, 95% CI, *p*-Value	β Estimate, 95% CI, *p*-Value
Age > 40	−0.467 (−0.742–−0.193) *p* = 0.001	−0.454 (−0.717–−0.192) *p* = 0.001	−0.537 (−0.815–−0.258) *p* < 0.001	−0.466 (−0.765–−0.167) *p* = 0.002	-
Female gender	0.067 (−0.201–0.336) *p* = 0.622	0.132 (−0.127–0.391) *p* = 0.317	0.069 (−0.205–0.344) *p* = 0.620	0.071 (−0.224–0.365) *p* = 0.638	0.073 (−0.223–0.369) *p* = 0.628
BMI (ref < 25) Overweight	−0.062 (−0.354–0.229) *p* = 0.676	−0.014 (−0.296–0.268) *p* = 0.923	−0.004 (−0.303–0.296) *p* = 0.981	−0.010 (−0.332–0.311) *p* = 0.950	−0.009 (−0.331 –0.313) *p* = 0.958
Obese/Extreme Obese	0.141 (−0.259–0.542) *p* = 0.490	0.262 (−0.114–0.638) *p* = 0.171	0.397 (−0.002–0.796) *p* = 0.051	0.317 (−0.111–0.746) *p* = 0.146	0.313 (−0.119–0.745) *p* = 0.155
Smoking	−0.408 (−0.694–−0.122) *p* = 0.005	−0.287 (−0.545–−0.028) *p* = 0.030	−0.378 (−0.652–−0.103) *p* = 0.007	−0.328 (−0.623–−0.033) *p* = 0.029	-
Hypertension	−0.106 (−0.619–0.406) *p* = 0.684	0.046 (−0.421–0.513) *p* = 0.847	0.074 (−0.422–0.570) *p* = 0.769	0.066 (−0.467–0.598) *p* = 0.809	0.068 (−0.465–0.602) *p* = 0.802
Immunosuppression	0.232 (−0.700–1.164) *p* = 0.626	0.335 (−0.505–1.175) *p* = 0.435	0.283 (−0.608–1.174) *p* = 0.534	0.243 (−0.714–1.199) *p* = 0.619	0.242 (−0.715–1.200) *p* = 0.620
Dyslipidemia	0.102 (−0.471–0.675) *p* = 0.728	0.294 (−0.221–0.809) *p* = 0.263	0.320 (−0.227–0.866) *p* = 0.252	0.286 (−0.301–0.872) *p* = 0.340	0.284 (−0.304–0.872) *p* = 0.344
Autoimmune	−0.273 (−0.742–0.195) *p* = 0.253	−0.267 (−0.700–0.166) *p* = 0.226	−0.372 (−0.831–0.087) *p* = 0.112	−0.292 (−0.785–0.201) *p* = 0.246	−0.291 (−0.785–0.203) *p* = 0.248
Interaction Effect					
(ref: Age ≤ 40 and No Smoking)					
Age ≤ 40 and Smoking					−0.289 (−0.820 –0.241) *p* = 0.285
Age > 40 and No Smoking					−0.451 (−0.797 –−0.106) *p* = 0.010
Age > 40 and Smoking					−0.796 (−1.177 –−0.416) *p* < 0.001

Notes: (1) Multivariate analysis for months one to four, (2) multivariate analysis for months four to nine, (3) multivariate analysis for months one to nine, and (4) multivariate analysis for months one to nine including interaction term. Abbreviations: CI: confidence interval, BMI: body mass index.

**Table 4:** Predictors of positive cellular immunity (logistic regression).

Variable	Univariate Analysis	Multivariate Analysis	
		(1)	(2)
	*n* = 189	*n* = 189	*n* = 189
	OR, 95% CI, *p*-Value	OR, 95% CI, *p*-Value	OR, 95% CI, *p*-Value
**Part A:** Cellular immunity OSP			
Age > 40	0.705 (0.390–1.276) *p* = 0.248	0.931 (0.458–1.894) *p* = 0.844	0.939 (0.463–1.906) *p* = 0.862
Female gender	0.565 (0.316–1.010) *p* = 0.054	0.477 (0.238–0.956) *p* = 0.037	0.515 (0.260–1.021) *p* = 0.057
BMI (ref < 25) Overweight	1.263 (0.675–2.363) *p* = 0.465	0.972 (0.465–2.033) *p* = 0.940	1.065 (0.514–2.209) *p* = 0.866
Obese/Extreme Obese	1.486 (0.631–3.496) *p* = 0.365	0.889 (0.325–2.434) *p* = 0.819	1.073 (0.404–2.852) *p* = 0.888
Smoking	0.645 (0.344–1.210) *p* = 0.172	0.720 (0.362–1.432) *p* = 0.349	0.731 (0.367–1.458) *p* = 0.374
Hypertension	1.303 (0.438–3.872) *p* = 0.634	1.519 (0.459–5.027) *p* = 0.493	1.419 (0.428–4.706) *p* = 0.567
Immunosuppression	0.419 (0.043–4.100) *p* = 0.455	0.235 (0.018–3.004) *p* = 0.265	0.369 (0.034–4.011) *p* = 0.412
Dyslipidemia	1.068 (0.314–3.630) *p* = 0.916	1.070 (0.266–4.301) *p* = 0.924	1.204 (0.308–4.704) *p* = 0.790
Autoimmune	0.884 (0.322–2.432) *p* = 0.812	1.293 (0.418–3.995) *p* = 0.656	1.151 (0.372–3.557) *p* = 0.807
Antibodies 1st month	1.003 (1.000–1.006) *p* = 0.044	1.001 (0.997–1.005) *p* = 0.775	
Antibodies 4th month	1.015 (1.004–1.025) *p* = 0.005	1.016 (1.002–1.031) *p* = 0.028	
Antibodies 9th month	1.007 (0.994–1.021) *p* = 0.276	1.005 (0.997–1.013) *p* = 0.224	
Ln (Antibodies 1st month)	1.645 (1.073–2.523) *p* = 0.023		1.139 (0.594–2.187) *p* = 0.695
Ln (Antibodies 4th month)	1.601 (1.135–2.258) *p* = 0.007		1.309 (0.694–2.470) *p* = 0.406
Ln (Antibodies 9th month)	1.545 (1.125–2.124) *p* = 0.007		1.253 (0.866–1.812) *p* = 0.232
**Part B:** Cellular immunity VSP			
Age > 40	0.649 (0.338–1.248) *p* = 0.195	0.677 (0.311–1.474) *p* = 0.326	0.719 (0.328–1.574) *p* = 0.409
Female gender	0.791 (0.416–1.506) *p* = 0.476	0.828 (0.385–1.778) *p* = 0.628	0.848 (0.396–1.815) *p* = 0.671
BMI (ref < 25) Overweight	1.330 (0.660–2.677) *p* = 0.425	1.265 (0.561–2.851) *p* = 0.570	1.332 (0.593–2.992) *p* = 0.488
Obese/Extreme Obese	1.257 (0.483–3.269) *p* = 0.639	0.833 (0.261–2.658) *p* = 0.757	1.006 (0.330–3.074) *p* = 0.991
Smoking	0.757 (0.372–1.539) *p* = 0.441	0.893 (0.413–1.931) *p* = 0.773	0.904 (0.416–1.963) *p* = 0.799
Hypertension	2.167 (0.713–6.584) *p* = 0.173	3.518 (1.077–12.102) *p* = 0.054	3.162 (0.920–10.860) *p* = 0.067
Immunosuppression	0.900 (0.091–8.855) *p* = 0.928	0.730 (0.053–10.020) *p* = 0.814	0.966 (0.078–11.892) *p* = 0.978
Dyslipidemia	1.016 (0.259–3.987) *p* = 0.982	1.086 (0.240–4.924) *p* = 0.915	1.158 (0.260–5.165) *p* = 0.847
Autoimmune	0.554 (0.152–2.012) *p* = 0.369	0.660 (0.157–2.768) *p* = 0.570	0.609 (0.145–2.546) *p* = 0.496
Antibodies 1st month	1.003 (0.999–1.006) *p* = 0.118	1.001 (0.997–1.005) *p* = 0.766	
Antibodies 4th month	1.011 (1.001–1.020) *p* = 0.026	1.010 (0.997–1.023) *p* = 0.123	
Antibodies 9th month	1.003 (0.999–1.006) *p* = 0.153	1.003 (0.999–1.006) *p* = 0.116	
Ln (Antibodies 1st month)	1.607 (0.982–2.631) *p* = 0.059		1.231 (0.580–2.614) *p* = 0.589
Ln (Antibodies 4th month)	1.534 (1.053–2.235) *p* = 0.026		1.113 (0.562–2.204) *p* = 0.759
Ln (Antibodies 9th month)	1.455 (1.076–1.968) *p* = 0.015		1.294 (0.909–1.843) *p* = 0.152

Notes: (1) Multivariate analysis with antibodies as continuous variables. (2) Multivariate analysis with antibodies as logarithmic variables. Abbreviations: OR: odds ratios, CI: confidence interval, BMI: Body mass index.

## Data Availability

All data are available upon reasonable request from the corresponding author.
